# The impact of *Sarcocystis* infection on lamb flavor metabolites and its underlying molecular mechanisms

**DOI:** 10.3389/fvets.2025.1543081

**Published:** 2025-03-28

**Authors:** Kang Zhang, Huan Teng, Li Zhu, Bin Ni, Lai Jiewei, Li Shanshan, Zhao Yunong, Xiao Guo, DanQu Lamu

**Affiliations:** ^1^College of Animal Science and Technology, Foshan University, Foshan, China; ^2^Institute of Animal Husbandry and Veterinary, Tibet Autonomous Regional Academy of Agricultural Sciences, Lhasa, China; ^3^Faculty of Animal Science and Technology, Yunnan Agricultural University, Kunming, China

**Keywords:** *Sarcocystis* infection, flavor metabolites, Tibetan sheep, lipid metabolism, transcriptomics, molecular mechanisms

## Abstract

**Introduction:**

Meat flavor is a critical factor for consumers to evaluate meat quality and a key determinant of its market value. *Sarcocystis* spp. are widely distributed parasitic protozoa that infect livestock, leading to reduced meat quality, fur, and fiber, and causing significant economic losses. However, most studies focus on the pathogenic mechanisms and epidemiological characteristics of *Sarcocystis*, with limited research on its specific impact on meat quality and flavor, particularly the underlying molecular regulatory mechanisms.

**Methods:**

This study investigated the effects of *Sarcocystis* infection on meat flavor and its molecular mechanisms in Tibetan sheep using flavor metabolite analysis and transcriptomic approaches. Tibetan sheep raised under uniform conditions were divided into four groups based on infection severity: normal, low-infection, moderate-infection, and high-infection. Leg muscle samples were collected for flavor metabolite analysis and transcriptome sequencing. Differentially expressed metabolites (DEMs) and differentially expressed genes (DEGs) were identified, and KEGG pathway enrichment analysis was performed to explore how *Sarcocystis* infection regulates gene expression, affecting lipid, amino acid, and energy metabolism, ultimately altering the production and accumulation of flavor metabolites.

**Results:**

The results showed that *Sarcocystis* infection significantly altered the composition of flavor metabolites in Tibetan sheep meat as infection severity increased. Phenolic and acidic metabolites were markedly upregulated, intensifying bitterness and sourness, while ketone and lactone metabolites were downregulated, reducing fatty and creamy aromas. Transcriptomic analysis identified 574 DEGs, including upregulated genes such as *MAPK12*, *COX6A2*, and *RXRA*, which are involved in lipid metabolism, fatty acid oxidation, and thermogenesis, and downregulated genes such as *COX2*, *COX3*, and *ADIPOQ*, which are associated with mitochondrial function and energy metabolism. These gene expression changes disrupted lipid and amino acid metabolism, leading to imbalances in the synthesis and accumulation of flavor compounds.

**Discussion:**

This study systematically revealed the significant effects of *Sarcocystis* infection on the meat flavor of Tibetan sheep and its underlying molecular mechanisms. The findings provide new insights into the metabolic regulation induced by parasitic infection and offer a theoretical basis for mitigating the adverse effects of *Sarcocystis* infection on meat quality.

## 1 Introduction

Meat flavor is a central criterion for consumers to assess meat quality and a critical determinant of market value (1). Lamb is widely appreciated for its distinctive flavor, which is mainly influenced by metabolites generated through biological processes like lipid metabolism, amino acid metabolism, and energy metabolism (2–4). These metabolites, such as ketones, aldehydes, acids, and phenols, play a direct role in shaping the aroma, taste, and overall flavor balance of the meat (5). However, meat flavor regulation is impacted not only by genetic, rearing, and environmental factors but also by the significant effects of diseases or parasitic infections (6).

*Sarcocystis* spp. are parasitic protozoans under the phylum Apicomplexa, named for the sarcocysts they develop in muscle or nerve tissues (7). Over 200 species of *Sarcocystis* have been identified, with most showing strong host specificity (species-specific for intermediate hosts and family-specific for definitive hosts) (8). *Sarcocystis* infection in livestock can cause a decline in the quality of meat, fur, and fibers, resulting in considerable economic losses (9). In humans, some *Sarcocystis* infections may lead to serious health problems, especially in individuals with weakened immune systems (10).

*Sarcocystis* infections are prevalent worldwide, especially in areas with intensive livestock farming. Recent years have seen substantial advancements in research on the molecular features and phylogenetic relationships of *Sarcocystis*. For instance, Gjerde (11) conducted molecular identification of *Sarcocystis* in reindeer and other hosts, discovering multiple new species and reassessing their phylogenetic relationships. Moreover, Cerqueira-Cezar et al. (12) expanded insights into parasite diversity and host adaptability by studying the morphology and molecular characteristics of *Sarcocystis* in Arctic foxes and moose. However, most studies to date have concentrated on the pathogenic mechanisms and epidemiology of *Sarcocystis* (13), while research on its impact on meat flavor and associated molecular regulatory mechanisms remains scarce.

This study seeks to systematically explore the impact of *Sarcocystis* infection on Tibetan sheep meat flavor and its underlying molecular mechanisms using flavor metabolite analysis and transcriptomics. Specifically, the study identified differentially expressed metabolites (DEMs) and differentially expressed genes (DEGs) and integrated KEGG pathway enrichment analysis to investigate how *Sarcocystis* infection modulates gene expression to influence lipid, amino acid, and energy metabolism, ultimately altering flavor metabolite production and accumulation.

The study integrates flavor metabolite analysis with transcriptomics to uncover the molecular mechanisms regulating meat flavor under *Sarcocystis* infection. This not only offers fresh insights into how parasitic infections regulate meat flavor but also provides critical evidence for alleviating the adverse effects of parasitic infections on meat quality.

## 2 Methods

### 2.1 Sample collection

This study involved 40 healthy adult Tibetan sheep raised under uniform conditions, categorized into four groups according to the level of *Sarcocystis* infection: control group (CK, *n* = 10), low-infection group (LK, *n* = 10), moderate-infection group (MK, *n* = 9), and high-infection group (HK, *n* = 9) (Supplementary Table S1).

Infection levels were determined using veterinary diagnoses and microscopic pathological examinations, supplemented by standardized microscopy to classify infection severity in unit-weight samples. All sheep were reared under the same conditions, and after slaughter, biceps femoris muscle samples were promptly collected. About 50 g of muscle tissue from each sheep was flash-frozen in liquid nitrogen and stored at −80°C for further flavor metabolite analysis and transcriptome sequencing. The animal protocol was reviewed and approved by the Experimental Animal Welfare and Animal Experiment Ethics Review Committee of Foshan University (approval number: FOSU202410-28). All procedures were conducted in strict accordance with the guidelines established by the Animal Use Committee of the Ministry of Agriculture of China, Beijing, to ensure the minimization of animal suffering throughout the study.

### 2.2 Determination and grouping criteria of Sarcocystosis infection levels

The determination of *Sarcocystis* infection levels was conducted with reference to the local standard, Technical Regulations for the Prevention and Control of Sarcocystosis in Cattle and Sheep, in the Tibetan region. The infection levels were primarily assessed through pathological diagnosis, microscopic examination, and species-specific PCR detection to ensure diagnostic accuracy and to exclude interference from other parasitic infections. Specifically, pathological diagnosis involved observing pathological characteristics of muscle tissues, such as white streaks or milky-white cysts distributed along the direction of muscle fibers, to preliminarily determine the infection status. Microscopic examination was performed by preparing compressed slides of muscle samples, which were cut into small strips along the direction of muscle fibers. The presence of *Sarcocystis* cysts (with shapes including spindle-shaped, cylindrical, or oval) was observed, and the number of cysts was recorded as the primary criterion for determining infection levels.

To further confirm the presence of *Sarcocystis* and to exclude other parasitic infections, such as *Toxoplasma gondii*, species-specific PCR detection was performed. DNA was extracted from muscle tissues using a commercial DNA extraction kit following the manufacturer’s protocol. PCR amplification targeted the small subunit ribosomal RNA (SSU rRNA) gene fragments, which are highly conserved and species-specific. To identify *Sarcocystis* and other parasites with similar symptoms, species-specific primers were used to amplify SSU rRNA gene fragments. The presence of *Sarcocystis* was confirmed by visualizing the expected amplicon size on a 1.5% agarose gel. To exclude *Toxoplasma gondii* infection, a separate PCR assay was conducted using primers specific to the SSU rRNA gene of *Toxoplasma gondii*. No amplification was observed for *Toxoplasma gondii* in any of the samples, confirming the absence of this parasite. These PCR-based diagnostic methods improved the sensitivity and accuracy of the diagnosis and ensured that the observed effects on meat quality were specifically attributable to *Sarcocystis* infection.

The detailed grouping criteria for infection levels were based on the infection count per unit weight of muscle (infection count per unit weight, infections/g). According to the infection levels, the samples were divided into four groups: normal group (CK), low infection group (LK), moderate infection group (MK), and high infection group (HK). The specific grouping criteria are as follows:

Normal group (CK): No infection detected (infection count per unit weight = 0).
Low infection group (LK): Infection count per unit weight less than 100 infections/g.
Moderate infection group (MK): Infection count per unit weight between 100 and 300 infections/g.
High infection group (HK): Infection count per unit weight greater than 300 infections/g.

### 2.3 Transcriptome analysis

Total RNA from muscle tissue was isolated using TRIzol reagent (Invitrogen, United States) (14) according to the kit’s protocol. The RNA concentration and purity were determined using a NanoDrop 2000 spectrophotometer (Thermo Fisher Scientific, United States) (15) by measuring the OD260/280 ratio, while RNA integrity was evaluated with an Agilent 2100 Bioanalyzer (Agilent Technologies, United States). Samples with RIN values ≥7.0 were selected for subsequent analysis (16). The transcriptome sequencing library was prepared using the Illumina TruSeq RNA Sample Preparation Kit (Illumina, United States), following standard protocols for mRNA enrichment, fragmentation, cDNA synthesis via reverse transcription, adapter ligation, and PCR amplification. The library quality was evaluated with an Agilent 2100 Bioanalyzer, and paired-end sequencing (PE150) was conducted on the Illumina NovaSeq 6000 platform (17).

Sequencing data quality was assessed with FastQC software (18), and Trimmomatic software (19) was used to remove adapter sequences and low-quality reads, resulting in high-quality clean data (Supplementary Table S2). The clean data were mapped to the sheep reference genome (ARS-UI_Ramb_v2.0) using HISAT2 software (20), and gene expression levels were quantified with StringTie software (21) (TPM values). Differentially expressed genes (DEGs) were screened using the DESeq2 software (22) with screening criteria of |log2FoldChange| >1 and *p* < 0.05 (Supplementary Table S8). Functional annotation of DEGs using GO (Gene Ontology) and KEGG (Kyoto Encyclopedia of Genes and Genomes) pathway enrichment analysis was conducted with the ClusterProfiler R package (23).

### 2.4 Quantitative real-time PCR validation

To validate the results of the sequencing analysis, RT-qPCR was performed on the selected genes. Total RNA was extracted from high-infection tissue samples using TRIzol reagent (Invitrogen, Carlsbad, CA, United States) and reverse-transcribed into cDNA using the TIANGEN Kit KR210831. The qPCR reactions were carried out using 2× SuperReal PreMix Plus (SYBR Green), with 2 μL of cDNA template and forward and reverse primers added. The total reaction volume was 20 μL, following the manufacturer’s instructions. Each qPCR experiment was performed in triplicate, and the average Ct value was used for subsequent analysis. The relative mRNA abundance was calculated using the $2^{-\Delta \Delta CT}$ method. The reaction conditions were as follows: 95°C for 15 min (1 cycle), followed by 95°C for 10 s and 60°C for 20 s (40 cycles). The sequences of the internal reference primers used for RT-qPCR are listed in Supplementary Table S15.

### 2.5 Flavor metabolites

#### 2.5.1 Detection for flavor metabolites

Frozen biceps femoris samples were powdered, and 1 g of the powder was weighed and mixed with 10 mL of pre-chilled extraction solvent. Following 30 min of ultrasonic extraction, the mixture was centrifuged at 4°C (12,000 rpm, 10 min), and the supernatant was used for flavor metabolite detection. The detection of flavor metabolites was conducted using gas chromatography-time-of-flight mass spectrometry (GC-TOFMS) (24).

Pretreatment of the experimental samples included a derivatization step to transform certain non-volatile compounds into volatile forms for easier detection. An internal standard normalization method was employed during detection to standardize the data and minimize the influence of systematic errors on the results. GC-TOFMS detection conditions were as follows: the chromatographic column was a DB-5MS capillary column (30 m × 0.25 mm × 0.25 μm), with an injection volume of 1 μL. High-purity helium was used as the carrier gas at a flow rate of 1.0 mL/min. The temperature program began at 40°C (held for 2 min), increased by 5°C/min to 250°C (held for 5 min). The ion source temperature was set at 230°C, and the scanning range was *m*/*z* 50–500.

#### 2.5.2 Analysis of the flavor metabolites

Following the acquisition of raw data, peak area information was extracted and preprocessed using the following steps: noise and outliers were filtered based on the interquartile range (IQR). Peaks extracted from the raw data were denoised, retaining only those with a relative standard deviation (RSD) below 30% and a detection rate exceeding 50%. The preprocessed peak area data were then used for further analysis. Metabolites were identified by matching with standard mass spectral libraries (NIST and HMDB) and relatively quantified using the internal standard method. The selection of differential metabolites was performed using both univariate and multivariate statistical analyses. Univariate analysis was performed using *t*-tests and analysis of variance (ANOVA), with an initial screening criterion of *p* < 0.05. On this basis, differential metabolites were further screened by combining multivariate analysis, using the variable importance in projection (VIP) value, with a threshold of VIP >1.

Multivariate statistical analysis employed orthogonal projections to latent structures-discriminant analysis (OPLS-DA). The OPLS-DA model’s quality was assessed using 7-fold cross-validation and 200 permutation tests, with *R*^2^ and *Q*^2^ values calculated to confirm the model’s explanatory and predictive capabilities. OPLS-DA analysis was conducted using SIMCA 14.1 software (25) to assess the differences in metabolite composition between groups with different infection levels and the control group. Significant differential metabolites were identified by integrating univariate and multivariate statistical analysis results and visualized through multiple methods, such as hierarchical clustering analysis, box plots, and K-means clustering analysis.

### 2.6 Association analysis of the DEMs and DEGs

Using flavor metabolite analysis, significantly differentially expressed metabolites (DEMs) between the infection group and the normal group were identified, while transcriptome analysis was used to identify significantly differentially expressed genes (DEGs) between the two groups. Pearson correlation analysis (26) was performed to associate differentially expressed genes and metabolites, identifying significantly correlated gene-metabolite pairs. Significantly correlated genes and metabolites were mapped onto KEGG pathways to detect notable differences in metabolic pathways between the infection and normal groups, investigating how *Sarcocystis* infection influences flavor metabolite production through gene expression regulation.

### 2.7 Statistical analysis

All experimental data were expressed as mean ± standard deviation (mean ± SD) and analyzed using SPSS 26.0 software (27). Differences between groups were analyzed using one-way analysis of variance (ANOVA) and independent sample *t*-tests, with the significance level set at *p* < 0.05. Multivariate analyses (PCA, PLS-DA) were performed using SIMCA 14.1 software.

## 3 Results

### 3.1 Analysis of the sample infection degree

**Figure 1:**
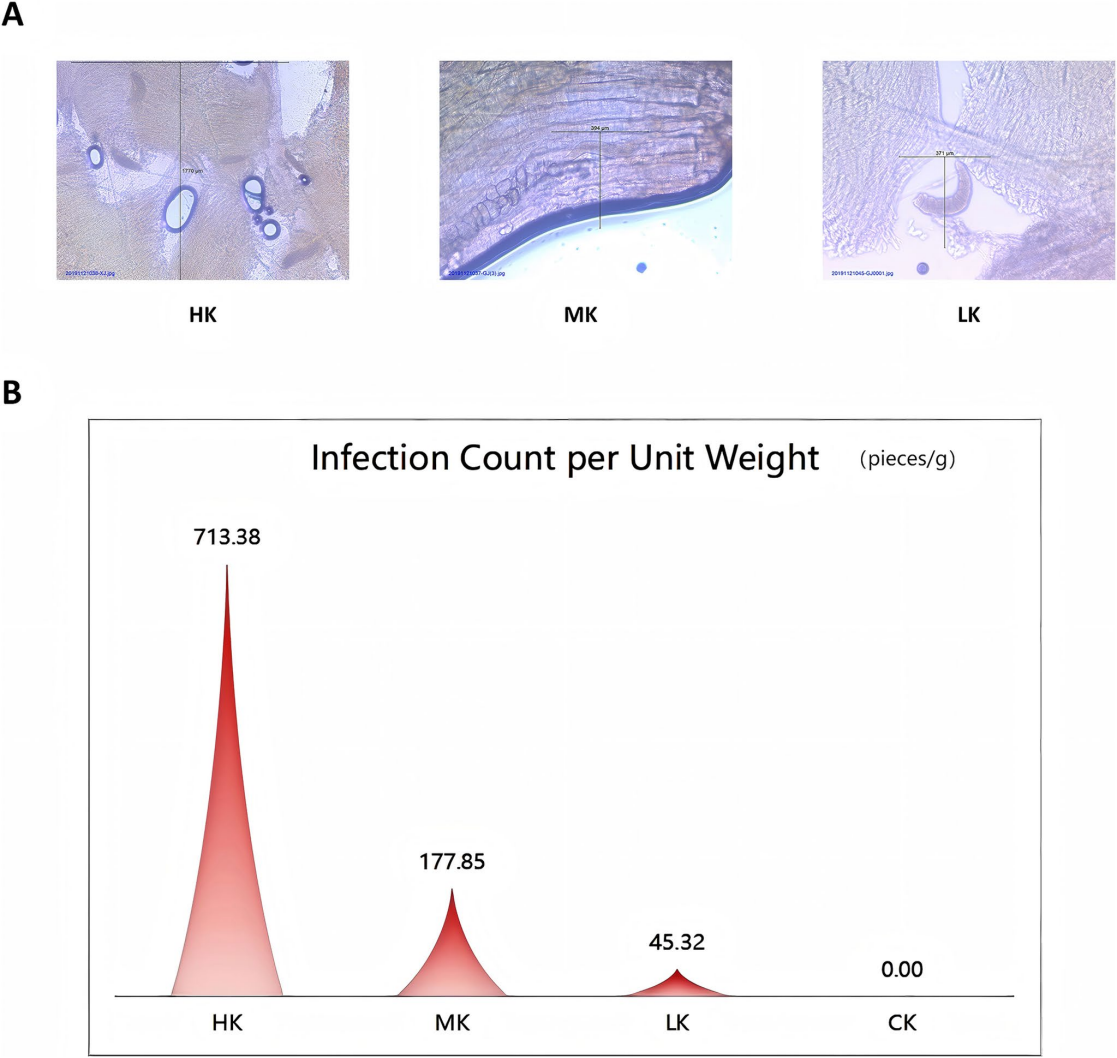
Overview of *Sarcocystis* infection levels. **(A)** Microscopic images showing different levels of *Sarcocystis* infection. **(B)** Comparison of infection counts per unit weight at varying levels of *Sarcocystis* infection.

To assess the infection level of *Sarcocystis* in Tibetan sheep, microscopic examination was performed on the collected samples to identify the degree of infection. The infection count per unit weight was calculated by quantifying the number of *Sarcocystis* cysts per unit weight in each sample group. Results indicated that there were significant differences in the infection count per unit weight across the groups. Specifically, the infection count per unit weight ranged from 320.00 to 1450.98 infections/g in the high infection group (HK), indicating severe infection levels. In the moderate infection group (MK), the infection count per unit weight ranged from 105.61 to 242.09 infections/g, reflecting a moderate level of infection. The low infection group (LK) exhibited infection counts ranging from 18.71 to 63.39 infections/g, representing a relatively mild infection level. In contrast, no infection was detected in the normal group (CK), with an infection count per unit weight of 0 infections/g (Figure 1B and Supplementary Table S1).

The microscopic field images provided a visual representation of the differences in *Sarcocystis* infection levels among the sample groups. The microscopic images revealed a gradual increase in cyst density from the low infection group (LK) to the high infection group (HK), validating the rationale behind the infection grouping (Figure 1A). These findings offer a solid foundation for further research into the impact of *Sarcocystis* infection on flavor metabolites and transcriptomic alterations.

### 3.2 Differential analysis of the flavor metabolites

#### 3.2.1 Overall distribution characteristics of the flavor metabolites

**Figure 2:**
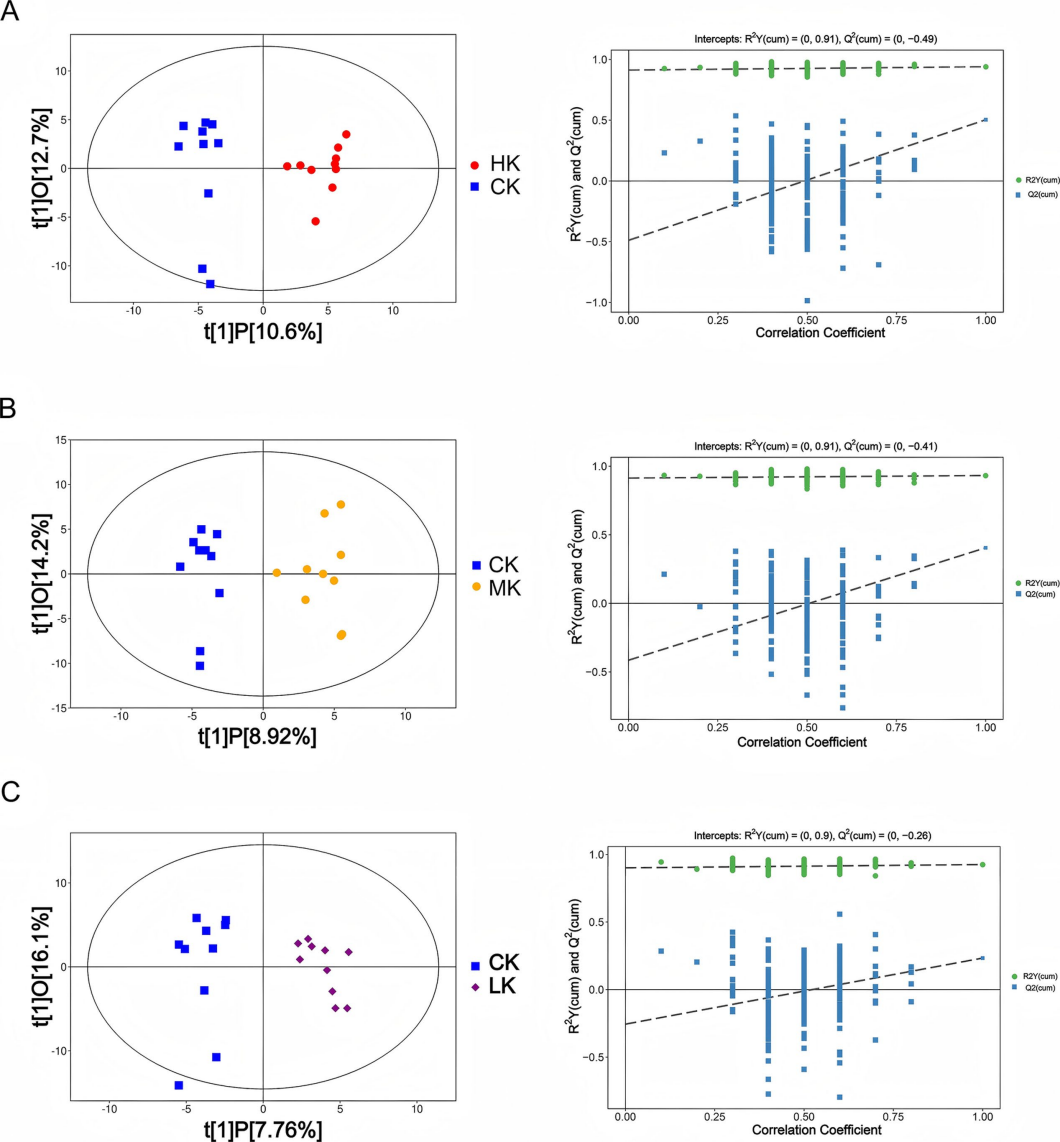
OPLS-DA model analysis for different comparison groups. **(A)** Left: OPLS-DA score scatter plot for CK group vs. HK group. Right: Permutation test results scatter plot for the OPLS-DA model (*n* = 200). **(B)** Left: OPLS-DA score scatter plot for CK group vs. MK group. Right: Permutation test results scatter plot for the OPLS-DA model (*n* = 200). **(C)** Left: OPLS-DA score scatter plot for CK group vs. HK group. Right: Permutation test results scatter plot for the OPLS-DA model (*n* = 200).

To investigate the overall distribution characteristics of flavor metabolites among different infection groups (CK, LK, MK, and HK), this study conducted multivariate statistical analysis of all identified metabolite peak data using orthogonal partial least squares discriminant analysis (OPLS-DA). The results demonstrated significant separation in metabolite composition between the CK group and the LK, MK, and HK groups. Additionally, the OPLS-DA permutation test results showed that the model exhibited strong explanatory and predictive capabilities (with high *R*^2^ and *Q*^2^ values), further validating the significance of metabolite differences among the infection groups (Figure 2).

#### 3.2.2 Screening of the differential metabolites

Differentially expressed metabolites (DEMs) between different infection groups were identified based on VIP values (VIP >1) and Log2 fold change values. The VIP value represents the contribution of metabolites to intergroup differences, while the Log2 fold change value indicates the extent of metabolite variation between groups (28).

**Figure 3:**
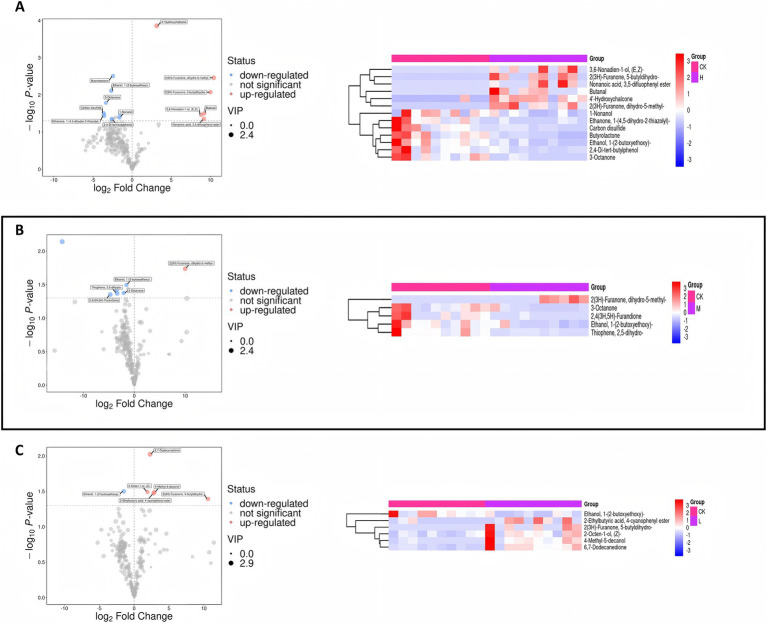
Differential metabolite statistics for the different control groups. **(A)** Left: Volcano plot for CK group vs. HK group. Point size represents VIP value (larger points indicate higher VIP), and color indicates regulation: red for upregulated, blue for downregulated, and gray for non-significant metabolites. Right: Hierarchical clustering analysis of differential metabolites. **(B)** Left: Volcano plot for CK group vs. MK group. Point size represents VIP value, and color indicates regulation: red for upregulated, blue for downregulated, and gray for non-significant metabolites. Right: Hierarchical clustering analysis of differential metabolites. **(C)** Left: Volcano plot for CK group vs. HK group. Point size represents VIP value, and color indicates regulation: red for upregulated, blue for downregulated, and gray for non-significant metabolites. Right: Hierarchical clustering analysis of differential metabolites.

**Figure 4:**
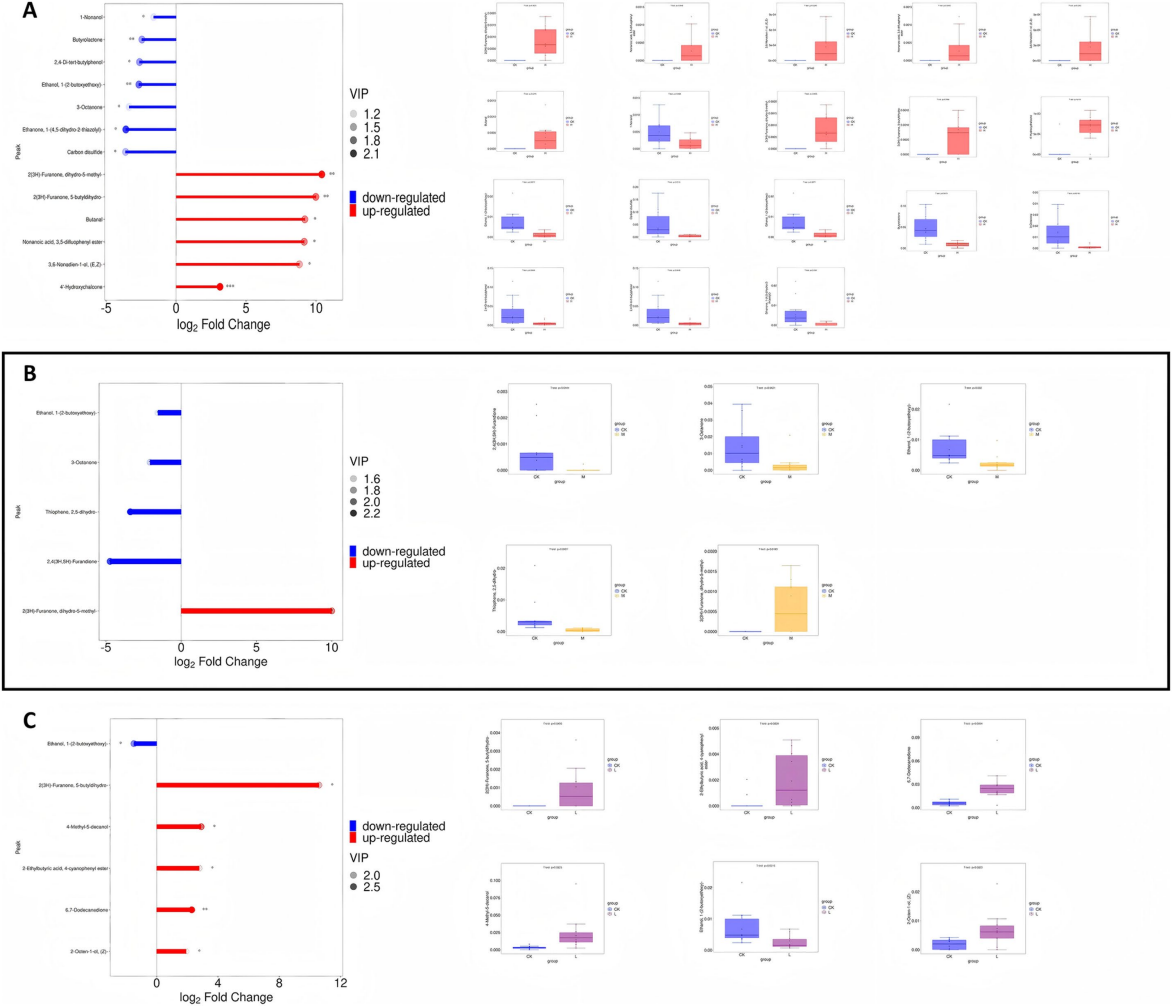
Analysis of changes in differential metabolites for different comparison groups (^*^p < 0.05, ^**^p < 0.01, and ^***^p < 0.01). **(A)** Left: Firewood chart analysis of changes in differential metabolites for CK group vs. HK group. Right: Box-plot analysis of quantitative values for each metabolite in each group. **(B)** Left: Firewood chart analysis of changes in differential metabolites for CK group vs. MK group. Right: Box-plot analysis of quantitative values for each metabolite in each group. **(C)** Left: Firewood chart analysis of changes in differential metabolites for CK group vs. HK group. Right: Box-plot analysis of quantitative values for each metabolite in each group.

In the comparison between the CK group and the HK group, 14 significantly different metabolites were identified, with six metabolites significantly upregulated and eight significantly downregulated (Supplementary Table S3 and Figure 3A). Specifically, butanal and furanone metabolites (2(3H)-furanone, dihydro-5-methyl-) were significantly upregulated, while ketone metabolites (3-octanone) and lactone metabolites (butyrolactone) were significantly downregulated. The significant upregulation of butanal and 2(3H)-furanone, in particular, may represent a key molecular basis for the flavor characteristic changes in the high infection group (Figure 4A).

In the comparison between the CK group and the MK group, seven significantly different metabolites were identified, with one metabolite significantly upregulated and six significantly downregulated (Supplementary Table S4 and Figure 3B). Furanone metabolites (2(3H)-furanone, dihydro-5-methyl-) were significantly upregulated, while ketones (3-octanone, 2,4(3H,5H)-furandione) and thiophene metabolites (thiophene, 2,5-dihydro-) were significantly downregulated, which may enhance caramel aroma while diminishing fatty and milky aromas (5, 29, 30) (Figure 4B). In the comparison between the CK group and the LK group, six significantly different metabolites were identified (Supplementary Table S5 and Figure 3C). Among them, furanone metabolites were significantly upregulated, while changes in alcohol metabolites suggested that the composition of flavor metabolites was already influenced to some extent, even in the low infection group (Figure 4C).

#### 3.2.3 Changing pattern of flavor metabolites

By screening and analyzing the differential metabolites between the CK group and the LK, MK, and HK groups, it was revealed that the degree of *Sarcocystis* infection significantly influenced the composition of flavor metabolites. With increasing infection severity (from CK to HK), certain metabolites exhibited significant trends. For instance, the relative abundance of furanone metabolites (2(3H)-furanone, dihydro-5-methyl-) increased progressively from −1.13 in the CK group to 1.29 in the HK group, indicating a significant upregulation with increasing infection severity (*p* < 0.01). This trend suggests that this metabolite may play a critical role in the flavor changes induced by infection. In contrast, the relative abundance of ketone metabolites (3-octanone and 6,7-dodecanedione) and lactone metabolites (butyrolactone) showed a decreasing trend with increasing infection severity. For example, the relative abundance of 3-octanone decreased from 0.23 in the CK group to −0.86 in the HK group (*p* < 0.05), while the relative abundance of butyrolactone decreased from 1.30 in the CK group to −1.14 in the HK group (*p* < 0.05). The downregulation of these metabolites may reduce the formation of fatty and milky aromas, potentially negatively impacting the texture and flavor profile of mutton (31–33).

Phenolic metabolites (2,4-di-tert-butylphenol) and acidic metabolites (2-ethylbutyric acid, 4-cyanophenyl ester) were significantly upregulated. For instance, the relative abundance of 2,4-di-tert-butylphenol increased from −0.48 in the CK group to 1.49 in the HK group (*p* < 0.01), while the relative abundance of 2-ethylbutyric acid, 4-cyanophenyl ester increased from −0.69 in the CK group to 1.47 in the HK group (*p* < 0.01). The upregulation of these metabolites may intensify bitterness and sourness, thereby disrupting the overall flavor balance. Additionally, alcohol metabolites (1-pentanol and 3-hexen-1-ol, formate, (Z)-) exhibited variable trends depending on the infection severity. For example, the relative abundance of 1-pentanol significantly increased from −0.40 in the CK group to 1.47 in the HK group (*p* < 0.01), whereas the relative abundance of 3-hexen-1-ol, formate, (Z)-decreased from −0.24 in the CK group to 0.01 in the HK group (*p* < 0.05). These changes reflect the complex role of alcohol metabolites in flavor modulation. These findings indicate that *Sarcocystis* infection alters the metabolic pathways responsible for the synthesis of flavor compounds, significantly affecting the sensory properties of mutton (33, 34) (Supplementary Table S6).

#### 3.2.4 Flavor classification of the differential metabolites

**Figure 5:**
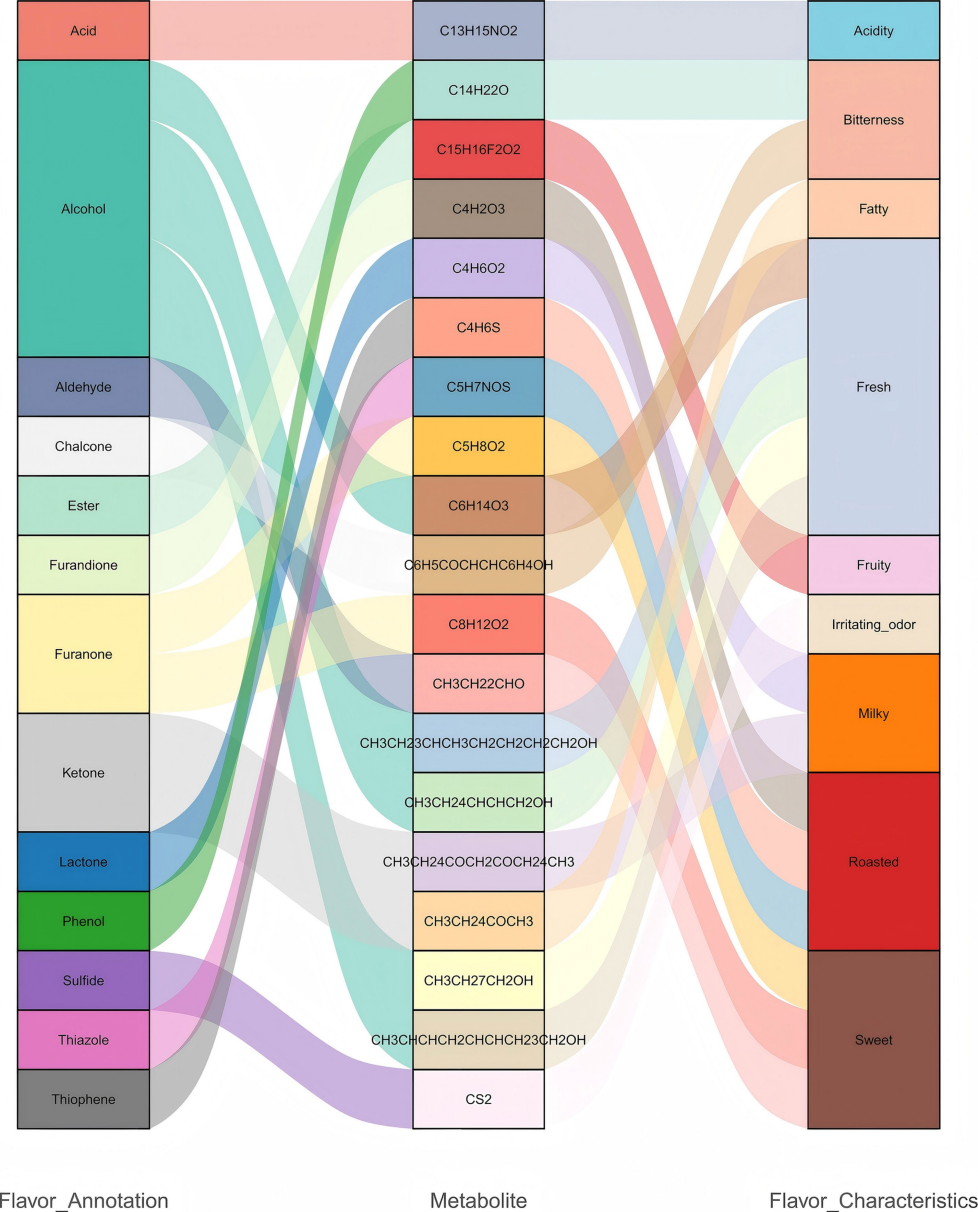
Relationship between metabolite classification and flavor annotation.

To further investigate the role of differential metabolites in flavor formation, this study integrated the differential metabolites identified between the CK group and the LK, MK, and HK groups and systematically annotated their flavor characteristics using relevant literature and databases (Supplementary Table S7). The results indicated that these metabolites were primarily distributed among the following chemical categories: aldehydes (butanal), ketones (3-octanone, 6,7-dodecanedione), alcohols (1-nonanol, 3,6-nonadien-1-ol, and 2-octen-1-ol), furanones (2(3H)-furanone, dihydro-5-methyl- and 2(3H)-furanone, 5-butyldihydro-), lactones (butyrolactone), phenolic metabolites (2,4-di-tert-butylphenol), and acidic metabolites (2-ethylbutyric acid, 4-cyanophenyl ester) (Figure 5). To examine the relative abundance trends of metabolites across different groups, *z*-score normalization was performed on the relative abundance of differential metabolites identified in all group comparisons, followed by K-means clustering analysis. The results revealed that with increasing infection severity (from LK to HK), phenolic metabolites (2,4-di-tert-butylphenol) and acidic metabolites (2-ethylbutyric acid, 4-cyanophenyl ester) were significantly upregulated, potentially intensifying bitterness and sourness, thus impacting the overall flavor balance. Additionally, ketone metabolites (3-octanone and 6,7-dodecanedione) and lactone metabolites (butyrolactone) were moderately downregulated, potentially reducing the formation of fatty and milky aromas, which could negatively impact the texture and flavor profile of mutton (Supplementary Table S6).

### 3.3 Transcriptome data analysis

#### 3.3.1 Sample clustering and grouping situation

To explore the molecular regulatory mechanisms of *Sarcocystis* infection on the flavor quality of Tibetan sheep meat and to uncover differences in gene expression levels between the high infection group (HK group) and the normal group (CK group), transcriptome analysis was conducted on foreleg muscle samples from 11 Tibetan sheep, with five samples in the HK group and six in the CK group. Principal component analysis (PCA) and hierarchical clustering analysis based on the top 500 differentially expressed genes were performed to systematically assess the overall gene expression patterns of the samples.

**Figure 6:**
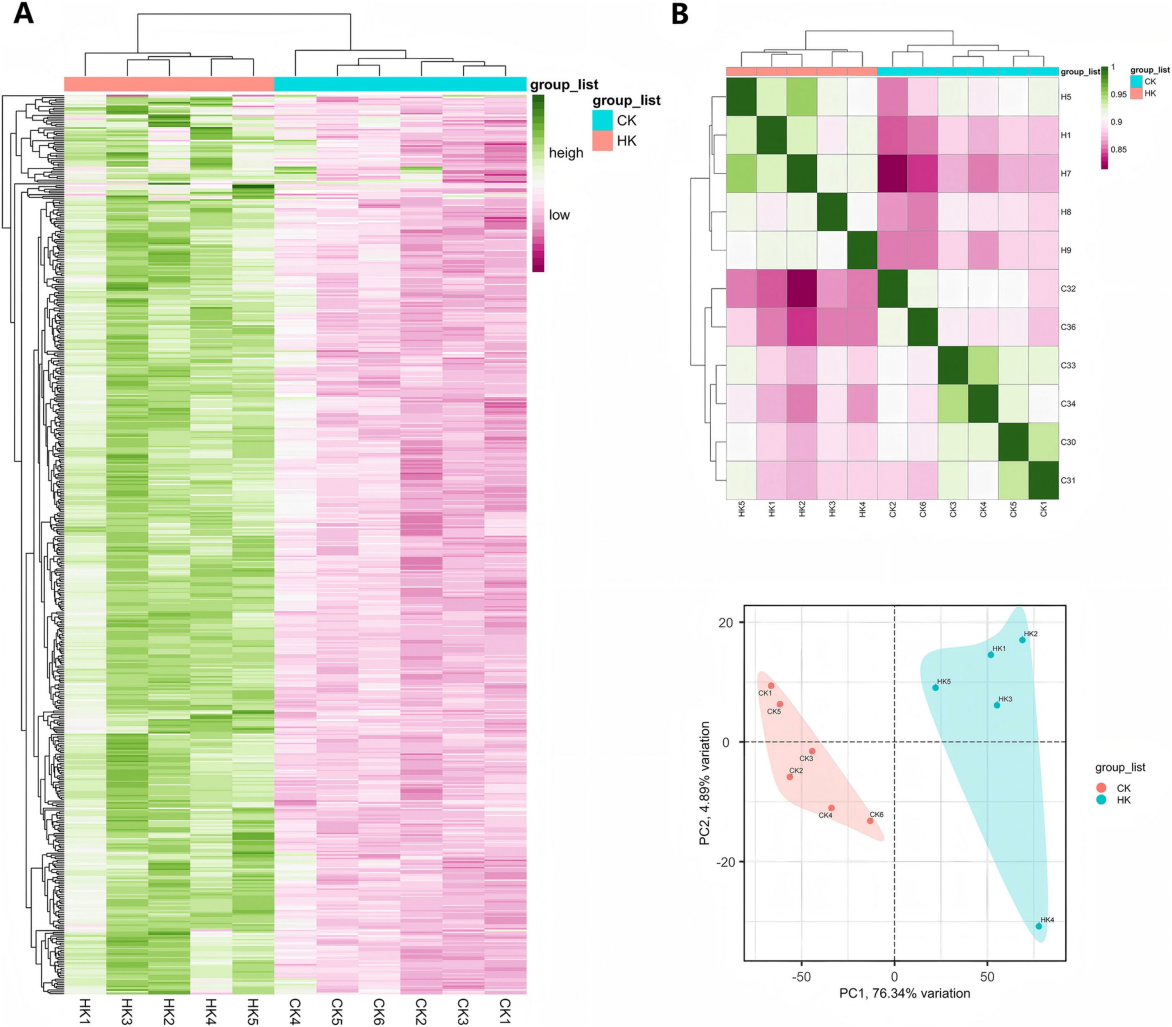
Analysis of differential genes and sample relationships. **(A)** Hierarchical clustering heatmap of the top 500 differential genes. **(B)** Sample correlation analysis and PCA (principal component analysis) results.

PCA results revealed that samples from the HK and CK groups were distinctly separated in the principal component space, indicating significant differences in gene expression levels between the two groups. Principal component 1 (PC1) and principal component 2 (PC2) accounted for a large proportion of the total variance, further validating the rationality and reliability of the sample grouping (Figure 6B). Additionally, hierarchical clustering analysis based on the top 500 differentially expressed genes further validated the significance of the sample grouping. Heatmap analysis revealed significant differences in the expression patterns of the top 500 differentially expressed genes between the two groups, with HK group samples showing highly consistent expression characteristics, whereas CK group samples exhibited a distinctly different expression pattern (Figure 6A). These findings further confirmed the significant differences in gene expression levels between the two groups, supporting subsequent differential gene screening and functional annotation analyses.

#### 3.3.2 Screening of the differentially expressed genes

**Figure 7:**
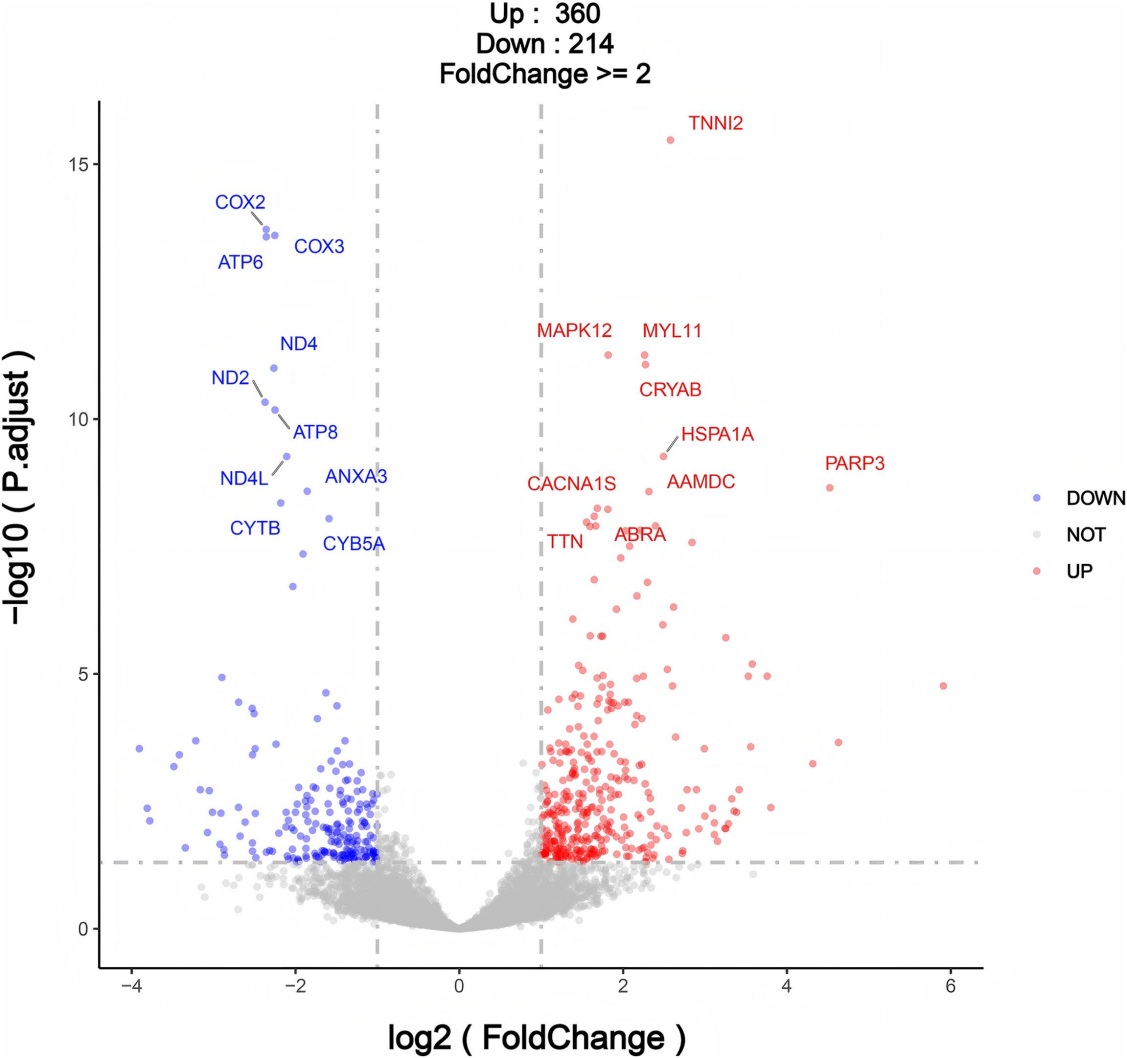
Volcano plot for differentially expressed genes (DEGs) in CK Group vs. HK group.

Differential expression analysis of genes (differentially expressed genes, DEGs) was conducted between the infection group (HK group) and the normal group (CK group) using transcriptome data. The screening criteria were set as: |log2FoldChange| >1 and *p* < 0.05. A total of 574 differentially expressed genes were identified, including 360 genes significantly upregulated in the infection group (HK group) and 214 genes significantly downregulated (Figure 7 and Supplementary Tables S9, S10). The identification of these differentially expressed genes provides a foundation for further exploration of the effects of *Sarcocystis* infection on gene regulatory networks and metabolic pathways.

#### 3.3.3 Quantitative real-time PCR (qPCR)

**Figure 8:**
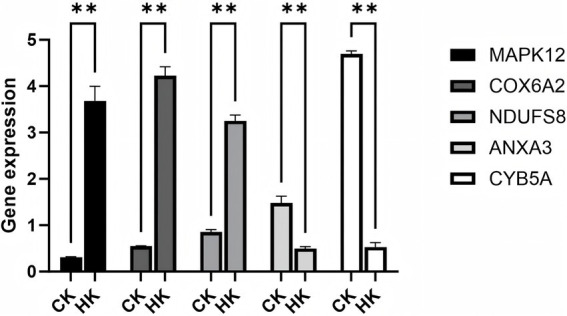
Validation of RNA-seq data using qPCR. ** Indicates *p* < 0.01, representing a highly significant difference.

We validated the RNA-seq data of five selected genes, including *MAPK12*, *COX6A2*, *NDUFS8*, *COX2*, and *ATP6*, and found that, compared to the normal group (CK), the expression levels of *MAPK12*, *COX6A2*, and *NDUFS8* were significantly upregulated in the high infection group (HK), while the expression levels of *COX2* and *ATP6* were significantly downregulated. These results indicate that identifying differentially expressed genes (DEGs) through transcriptomic data is both effective and reliable (Figure 8).

#### 3.3.4 Enrichment analysis of the differentially expressed genes

KEGG pathway enrichment analysis was conducted on the DEGs, identifying significantly enriched pathways (*p* < 0.05) (35). The results indicated that upregulated DEGs were significantly enriched in several pathways associated with the muscle cell cytoskeleton, energy metabolism, protein processing, and metabolic regulation. Among muscle function-related pathways, upregulated DEGs were significantly enriched in the cytoskeleton in muscle cells and cardiac muscle contraction. In energy metabolism-related pathways, upregulated DEGs were significantly enriched in non-alcoholic fatty liver disease, oxidative phosphorylation, and thermogenesis. Furthermore, upregulated DEGs were significantly enriched in pathways such as protein processing in the endoplasmic reticulum and nucleotide metabolism, indicating that infection may influence muscle cell homeostasis and function by regulating protein folding and metabolite synthesis (36–39) (Supplementary Table S11).

It is noteworthy that upregulated DEGs were also enriched in pathways associated with various diseases, such as Alzheimer disease, Prion disease, and pathways of neurodegeneration—multiple diseases. These findings suggest that *Sarcocystis* infection may indirectly influence muscle tissue function and meat flavor by modulating molecular mechanisms associated with neural and metabolic processes (40).

KEGG pathway enrichment analysis was conducted on the downregulated DEGs, identifying significantly enriched pathways (*p* < 0.05). The results indicated that downregulated DEGs were significantly enriched in several pathways associated with energy metabolism, oxidative stress, neurodegenerative diseases, and cell signaling. Among energy metabolism-related pathways, downregulated DEGs were significantly enriched in oxidative phosphorylation, thermogenesis, and non-alcoholic fatty liver disease (41, 42). In pathways related to oxidative stress and neurodegenerative diseases, downregulated DEGs were significantly enriched in Prion disease, Huntington disease, Parkinson disease, Alzheimer disease, and pathways of neurodegeneration—multiple diseases. Furthermore, downregulated DEGs were significantly enriched in pathways such as chemical carcinogenesis—reactive oxygen species and retrograde endocannabinoid signaling, further highlighting the importance of oxidative stress in *Sarcocystis* infection (43, 44). Among pathways related to cell signaling and tissue function, downregulated DEGs were significantly enriched in focal adhesion and cardiac muscle contraction, indicating that *Sarcocystis* infection may influence the structure and function of muscle tissue by modulating the expression of genes associated with cell adhesion and muscle contraction (Supplementary Table S12).

It is noteworthy that downregulated DEGs were also enriched in pathways associated with infection and immunity, such as influenza A and toxoplasmosis, implying that *Sarcocystis* infection may affect host immune responses and metabolic adaptation through the regulation of immune-related pathways (45).

Furthermore, GO functional enrichment analysis (*p* < 0.01) was conducted on the differentially expressed genes (DEGs). The results indicated that upregulated DEGs were primarily enriched in biological processes associated with energy metabolism and redox reactions, including oxidative phosphorylation, electron transport chain, cellular respiration, and hypoxia response. Downregulated DEGs were significantly enriched in processes related to muscle development, muscle contraction, protein folding, and stress responses, such as muscle cell development, striated muscle contraction, protein folding, and response to heat stimulus. The GO enrichment results further supported that *Sarcocystis* infection may influence the formation of meat flavor by modulating GO pathways associated with energy metabolism (oxidative phosphorylation, electron transport chain) and redox reactions. Additionally, its regulation of processes like muscle development, contraction, and protein folding may further modify the texture and flavor properties of the meat (Supplementary Tables S13, S14).

#### 3.3.5 Integrated analysis of flavor metabolites and transcriptomics

**Figure 9:**
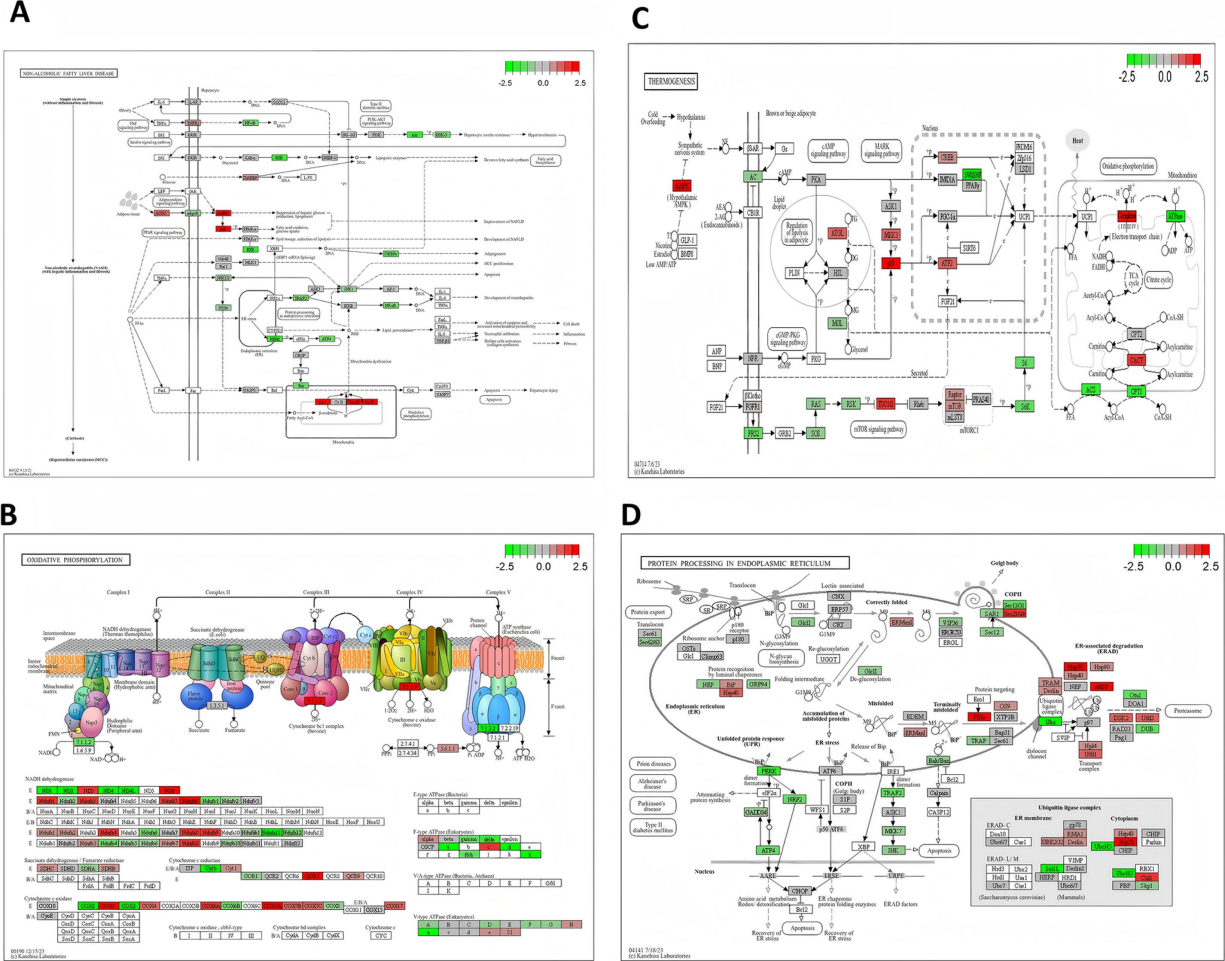
KEGG pathway map highlighting upregulated and downregulated genes. This figure shows a KEGG pathway map with upregulated (red) and downregulated (green) genes, illustrating their direct or indirect effects on metabolites and highlighting key metabolic pathways influenced by differential genes.

The flavor of meat is mainly influenced by the regulation of lipid metabolism, amino acid metabolism, and energy metabolism (4, 46). To explore the molecular mechanisms by which *Sarcocystis* infection affects lamb flavor, this study performed a combined analysis of differentially expressed genes (DEGs) and their significantly enriched KEGG pathways. Particular attention was given to key pathways involved in lipid metabolism, energy metabolism, and protein processing, as these pathways are closely linked to the generation and accumulation of flavor compounds. The KEGG pathways analyzed included the lipid metabolism-related pathway “Non-alcoholic fatty liver disease (NAFLD, 04932),” the energy metabolism-related pathways “Oxidative phosphorylation (00190)” and “Thermogenesis (04714),” as well as the protein processing and amino acid metabolism-related pathway “Protein processing in endoplasmic reticulum (04141)” (Figure 9). Differentially expressed genes within these pathways may directly or indirectly regulate lipid, amino acid, and energy metabolism, thus affecting the synthesis and accumulation of flavor compounds (5).

## 4 Discussion

This study systematically uncovered the significant effects of *Sarcocystis* infection on the meat flavor of Tibetan sheep and its underlying molecular mechanisms. Using flavor metabolite analysis and transcriptomic research, we discovered that the progression of infection significantly modified the composition of lamb flavor metabolites and gene expression profiles. These alterations not only influenced the sensory qualities of the meat but also highlighted the intricate mechanisms of host metabolic regulation induced by parasitic infection.

### 4.1 Comparison of the effects of *Sarcocystis* infection with other parasitic infections

Parasitic infections, such as those caused by *Sarcocystis*, *Toxoplasma gondii*, and *Trichinella spiralis*, share similarities in their transmission routes, host–parasite interactions, and impacts on host metabolism and meat quality. However, these parasites exhibit significant differences in pathogenicity, life cycles, and the extent of their physiological effects on the host.

*Toxoplasma gondii* is a protozoan parasite capable of infecting a wide range of warm-blooded animals, including humans. Its tissue cysts are commonly found in edible parts of animals such as pigs, sheep, and goats, where they can persist for extended periods (47). Studies have shown that *T. gondii* infection induces metabolic alterations, including oxidative stress and inflammation, which may indirectly affect meat quality by altering muscle composition and increasing the risk of spoilage. Furthermore, *T. gondii* infection is closely associated with public health concerns, such as congenital toxoplasmosis and neuropsychiatric disorders (48, 49). *Trichinella spiralis*, a nematode parasite, directly invades muscle tissue during its larval stage, forming “nurse cells” within the host. Infection can lead to severe myositis and metabolic disturbances, including increased energy expenditure and muscle inflammation. Research has demonstrated that *T. spiralis* infection significantly reduces meat quality by damaging muscle fiber structure, decreasing tenderness, and altering the biochemical composition of the meat. In heavily infected animals, the presence of larvae can severely compromise the commercial value of the meat (50–52).

In contrast, the findings of this study reveal that *Sarcocystis* infection exerts more complex effects on host metabolism and meat quality. *Sarcocystis* infection not only increases the accumulation of phenolic and acidic metabolites through lipid metabolism disorders and oxidative stress but also reduces the production of lipid-derived flavor compounds by inhibiting fatty acid oxidation and protein processing. Similar to *T. gondii*, *Sarcocystis* forms tissue cysts in muscle. Previous studies have reported that certain species of *Sarcocystis* (*Sarcocystis fayeri* in horses and *Sarcocystis suihominis* in pigs) can induce myositis and metabolic disturbances, including oxidative stress and inflammation (53, 54). These changes disrupt the balance of meat flavor and significantly reduce sensory quality. Additionally, *Sarcocystis* infection markedly alters the expression of genes associated with energy metabolism and muscle function, further exacerbating muscle tissue damage and the deterioration of meat quality.

### 4.2 Alterations in flavor metabolites and their impacts

As the severity of infection increased, phenolic and acidic metabolites (2,4-di-tert-butylphenol and 2-ethylbutyric acid, 4-cyanophenyl ester) were significantly upregulated, intensifying bitterness and sourness (55); meanwhile, ketone and lactone metabolites (3-octanone and butyrolactone) were significantly downregulated, diminishing fatty and creamy aromas (56, 57). These alterations disrupted the overall flavor balance, leading to a significant decline in the sensory quality of the meat. The buildup of phenolic and acidic metabolites might be linked to lipid metabolism disorders and oxidative stress, whereas the decrease in ketone and lactone metabolites could be attributed to the overactivation of fatty acid oxidation. These findings indicate that *Sarcocystis* infection modifies the synthesis and accumulation of flavor metabolites through multi-layered metabolic regulatory mechanisms, either directly or indirectly.

These findings suggest that *Sarcocystis* infection influences the flavor profile of Tibetan sheep meat by modulating the production of flavor metabolites. This aligns with existing research, which suggests that parasitic infections may indirectly influence lipid and amino acid metabolism by modifying the host’s metabolic state and immune response, ultimately resulting in changes to flavor metabolites (45).

### 4.3 Alterations in gene expression patterns and their mechanisms

Transcriptomic analysis revealed that *Sarcocystis* infection significantly modified gene expression patterns associated with energy metabolism, lipid metabolism, and protein processing. Upregulated genes were prominently enriched in pathways associated with mitochondrial function, fatty acid oxidation, and redox reactions, including the electron transport chain, ATP synthesis-coupled electron transport, and cellular respiration. This indicates that the infection may influence the synthesis and accumulation of lipid metabolites by promoting energy metabolism and fatty acid oxidation (58, 59). Furthermore, upregulated DEGs were significantly enriched in pathways linked to protein processing and redox reactions, including protein processing in the endoplasmic reticulum and cellular respiration. The activation of these pathways reflects the host cells’ response to infection stress and their efforts to maintain cellular homeostasis. Conversely, downregulated genes were prominently enriched in pathways associated with muscle development and function, including sarcomere organization, myofibril assembly, and striated muscle cell differentiation. Such alterations in gene expression patterns exacerbate muscle functional damage, indirectly resulting in a deterioration of the muscle’s physical and flavor properties, ultimately impacting the texture and taste of the meat (60, 61). It is noteworthy that DEGs were enriched in pathways associated with multiple diseases (toxoplasmosis, Alzheimer’s disease, Prion disease), indicating that *Sarcocystis* infection may indirectly influence muscle tissue function and flavor traits through the regulation of neural and metabolism-related molecular mechanisms.

Additionally, the functional alterations of specific genes provided further insights into the underlying molecular mechanisms. Within the *NAFLD* pathway, upregulated genes like *MAPK12*, *COX6A2*, and *RXRA* may facilitate the production of lipid-derived volatile flavor compounds by boosting fatty acid oxidation, lipid metabolism, and thermogenesis. The reduced expression of downregulated genes like *COX2*, *COX3*, and *ADIPOQ* may inhibit fatty acid oxidation, leading to a decrease in the accumulation of lipid-derived flavor compounds. Such metabolic imbalance leads to a decline in lipid-derived flavor compounds, diminishing the development of fatty and creamy aromas. Moreover, as the infection severity increased, phenolic metabolites (2,4-di-tert-butylphenol) and acidic metabolites (2-ethylbutyric acid, 4-cyanophenyl ester) were significantly upregulated, potentially linked to oxidative stress induced by lipid metabolism disorders. The buildup of phenolic and acidic metabolites may intensify bitterness and sourness, disrupting the overall flavor balance.

Furthermore, the decreased expression of downregulated genes *ATP6* and *ND4* may result in mitochondrial impairment and energy metabolism disruption. These metabolic disruptions may suppress the metabolic activity of fatty acids and amino acids, thereby decreasing the production of flavor compounds. Downregulated genes like *COX7A2L* and *ACTB* may indirectly influence the release of flavor compounds by impairing protein processing and muscle function.

### 4.4 Potential mitigation strategies

Given that parasitic infections often exert adverse effects on the host through oxidative stress and inflammatory mechanisms, dietary and pharmacological interventions are considered potential effective strategies to mitigate these negative impacts. Studies have shown that supplementation with antioxidants (vitamin E, selenium, or polyphenols) can significantly reduce oxidative stress and improve the meat quality of infected animals (62). For instance, dietary selenium has been demonstrated to enhance the host’s immune response and reduce tissue damage caused by *Toxoplasma gondii* infection. Additionally, diets rich in omega-3 fatty acids, due to their anti-inflammatory properties, may help alleviate muscle inflammation induced by *Trichinella spiralis* and *Sarcocystis* infections (63–65).

Pharmacological antioxidants, such as N-acetylcysteine or glutathione precursors, have also shown potential in mitigating oxidative damage associated with parasitic infections. These compounds may protect muscle tissue integrity and improve meat quality by reducing lipid peroxidation and protein oxidation (65, 66). From an environmental and husbandry perspective, controlling host populations and improving feed hygiene can effectively reduce environmental contamination with *T. gondii* oocysts and *Sarcocystis* sporocysts. Furthermore, heating meat to appropriate temperatures can effectively inactivate tissue cysts and larvae of *T. gondii*, *T. spiralis*, and *Sarcocystis*. However, this approach is limited to post-slaughter meat processing and cannot address metabolic disturbances or meat quality changes in live animals prior to slaughter caused by parasitic infections. Therefore, a comprehensive approach combining dietary, pharmacological, and environmental control measures may represent a more effective mitigation strategy.

### 4.5 Significance and future perspectives

Although previous studies have investigated the mechanisms underlying meat flavor formation in livestock and the pathogenic mechanisms and phylogenetic traits of *Sarcocystis* infection, no research has systematically elucidated the specific effects of *Sarcocystis* infection on the meat flavor of Tibetan sheep and its underlying molecular mechanisms. This study focused on the effects of *Sarcocystis* infection on meat flavor and, through a combination of flavor metabolite analysis and transcriptomic techniques, sought to unravel the molecular regulatory mechanisms by which infection influences the production of flavor metabolites, highlighting the profound effects of parasitic infection on the flavor and quality of Tibetan sheep meat. This study not only offers a novel perspective for understanding the intricate interactions between parasites and hosts but also provides a theoretical foundation for reducing the adverse effects of parasitic infections on meat quality.

### 4.6 Limitations

This study revealed the impact of *Sarcocystis* infection on the flavor of Tibetan sheep meat and its underlying molecular mechanisms; however, several limitations should be acknowledged. First, the observed variations in flavor metabolites may have been influenced by multiple factors, such as rearing conditions and environmental influences, which were not fully controlled or accounted for in this study. Additionally, the dynamic effects of infection duration on meat flavor were not investigated, leaving a gap in understanding the temporal mechanisms by which parasitic infection affects flavor development over time.

Second, this study did not directly address the role of oxidative stress in parasitic infection. Oxidative stress is a well-known factor in parasitic infections and may influence the synthesis of flavor compounds by regulating lipid peroxidation and amino acid metabolism. However, due to the limitations of the study design, quantitative detection of oxidative stress markers, such as through ELISA, was not included. This omission restricts a comprehensive understanding of the relationship between oxidative stress and flavor metabolism under parasitic infection.

Future research should aim to overcome these limitations by exploring the specific functions of differentially expressed genes and metabolites, as well as their interaction mechanisms. Functional genomics approaches, such as gene knockout or overexpression experiments, could be employed to validate the roles of key genes in flavor metabolism. Furthermore, incorporating quantitative analyses of oxidative stress markers (ELISA or similar methods) would provide deeper insights into the relationship between oxidative stress and the synthesis of flavor compounds. These efforts would contribute to a more thorough understanding of the regulatory mechanisms by which parasitic infections influence meat flavor and offer scientific support for mitigating the adverse effects of such infections on meat quality.

## 5 Conclusion

This study uncovered the significant impact of *Sarcocystis* infection on the meat quality and flavor of Tibetan sheep and its underlying molecular mechanisms. With the progression of infection, the composition of lamb flavor metabolites underwent significant changes: phenolic and acidic metabolites were markedly upregulated, intensifying bitterness and sourness, whereas ketone and lactone metabolites were downregulated, diminishing fatty and creamy aromas, ultimately disrupting the overall flavor balance. Transcriptomic analysis demonstrated that the infection significantly modified gene expression patterns associated with energy metabolism, lipid metabolism, and protein processing. Upregulated genes promoted mitochondrial function and fatty acid oxidation, whereas downregulated genes inhibited muscle development and function, further influencing the physical properties and flavor characteristics of the meat. By integrating flavor metabolite analysis with transcriptomics, we identified that changes in the expression of differential genes, including *MAPK12*, *COX6A2*, *RXRA*, *COX2*, *COX3*, *ADIPOQ*, *ATP6*, *ND4*, and *ACTB*, could directly or indirectly affect the production of flavor metabolites via pathways related to lipid metabolism, amino acid metabolism, and energy metabolism, mediated by multi-layered molecular regulatory mechanisms. This study offers a novel perspective on the regulation of meat flavor by parasitic infection and provides valuable insights into alleviating the adverse effects of parasitic infection on meat quality.

## Data Availability

The datasets presented in this study can be found in online repositories. The names of the repository/repositories and accession number(s) can be found at: https://www.ncbi.nlm.nih.gov, accession number: PRJNA1238310.
